# A Quantification of Target Protein Biomarkers in Complex Media by Faradaic Shotgun Tagging

**DOI:** 10.1021/acs.analchem.1c03695

**Published:** 2022-01-27

**Authors:** Mohamed Sharafeldin, Felix Fleschhut, Timothy James, Jason J. Davis

**Affiliations:** † Department of Chemistry, University of Oxford South Parks Road, Oxford OX1 3QZ, U.K.; ‡ Department of Clinical Biochemistry, John Radcliffe Hospital Oxford University Hospitals NHS Foundation Trust, Oxford OX3 9DU, U.K.

## Abstract

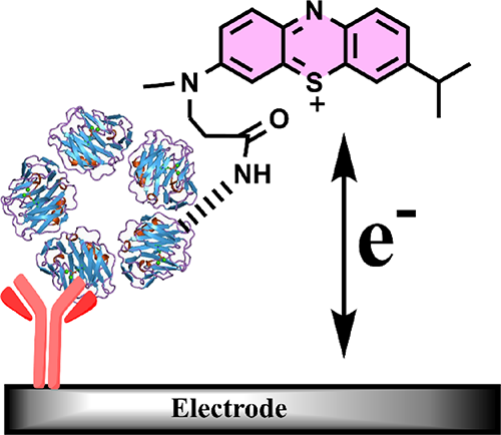

The progressive emergence of protein biomarkers promises a revolution in the healthcare industry and a shift of focus from disease management to much earlier intervention. Here, we introduce a facile shotgun tagging of ensemble proteins in clinically relevant media prior to specific target capture at antibody-modified electrodes. This facilitates a convenient voltammetric quantification of markers down to sub-pg/mL levels and across several orders of concentration. A translation of the methodology to an automated microfluidic platform enables marker quantification from 25 μL of sample in less than 15 min, demonstrated here with a simultaneous assaying of CRP and cardiac troponin I (cTnI). The assays show a good correlation with a standard immunoassay when applied to real patient serum samples. The platform is simple, generic, highly sensitive and requires no secondary labeling/binding or amplification.

## Introduction

The quantification of proteins in biological samples has emerged as an indispensable tool in clinical diagnostics, drug discovery, and the evaluation of therapeutic intervention.^1^ In particular, a quantified assessment of multiple protein markers has been associated with an improved understanding of abnormal metabolic states, physiological conditions, and diseases.^2−4^ Among the currently utilized approaches for target protein quantification, the enzyme-linked immunosorbent assay (ELISA) is widely regarded as a benchmark in terms of specificity and sensitivity. In its standard form, it utilizes paired antibodies along with an attached enzyme label to generate a colorimetric quantifiable readout and is available in a semiautomated format with clinical analyzers.^5^ Although ELISA derivatives have evolved much in recent years (such as the single-molecular arrays (Simoa)^6^ or electrochemiluminescence-based immunoassays, e.g., the Meso Scale Discovery (MSD) platform with its inherent amplification routinely enabling sub-pg/mL target detection levels), it also requires centralized laboratory settings, expensive hardware, demanding technical manipulation, and relatively long assay times (6–8 h for conventional ELISA), greatly limiting its translation to resource-limited or “point-of-care” (POC) settings.^7^ In contrast, electrochemical biosensors, which provide comparable levels of sensitivity, offer the possibility of developing cheap and highly scalable platforms, making their application in early diagnostics and health care solutions accessible to a much wider range of environments and proportions of the population. A diverse range of microfluidic electrochemical immunoassay protein analysis strategies have been investigated as cost-effective POC alternatives.^8,9^ These can be collectively divided into those that are sandwich in nature and those that are label-free.^10^ Although the former present high sensitivities derived from enzyme and/or nanoparticle labels^11,12^ as with ELISA, they are invariably associated with label generation as well as multiple washing and incubation steps, increasing complexity, required end-user intervention, time, and cost.^13^ Specifically derived secondary ligands—often antibodies conjugated with a detectable probe—are, in addition, needed for each target molecule (further increasing complexity). Label-free electrochemical methodologies aim to directly transduce target protein capture at an electrode-confined recognition element into a measurable signal, such as that detected by interfacial impedance or capacitance, and typically rely on specifically engineered electrode interfaces to maximize signal specificity in complex media.^14−16^

Ensemble protein labeling (shotgun tagging) has been commonly employed in mass spectrometry (MS) quantification, where target peptides are derivatized with specific isotopic masses^17^ or isobaric mass tags.^18,19^ A diverse range of MS applications utilizing such tagging techniques have been introduced in the last decade to improve sensitivity and to support, for example, the analysis of protein interactions.^20^ MS methods, of course, remain expensive and are very often semiquantitative only, operationally demanding, and far from scalable.^21^ Ensemble protein labeling with fluorescent or chemiluminescent probes has also been widely associated with cell-based protein imaging,^22^ where a range of aryl-diazonium,^23^ azlactone,^24^ vinyl sulfone,^25^ and NHS-ester/isothiocyanate bioconjugation strategies have been applied.^26^ Among these, succinimidyl ester approaches, operating with good levels of selectivity under conditions of neutral pH with easily stored reagents, are ubiquitous,^18,19,27,28^ targeting primary amine groups of the N termini of any polypeptide chain or solvent-exposed lysine residues.^29^

**Scheme 1:**
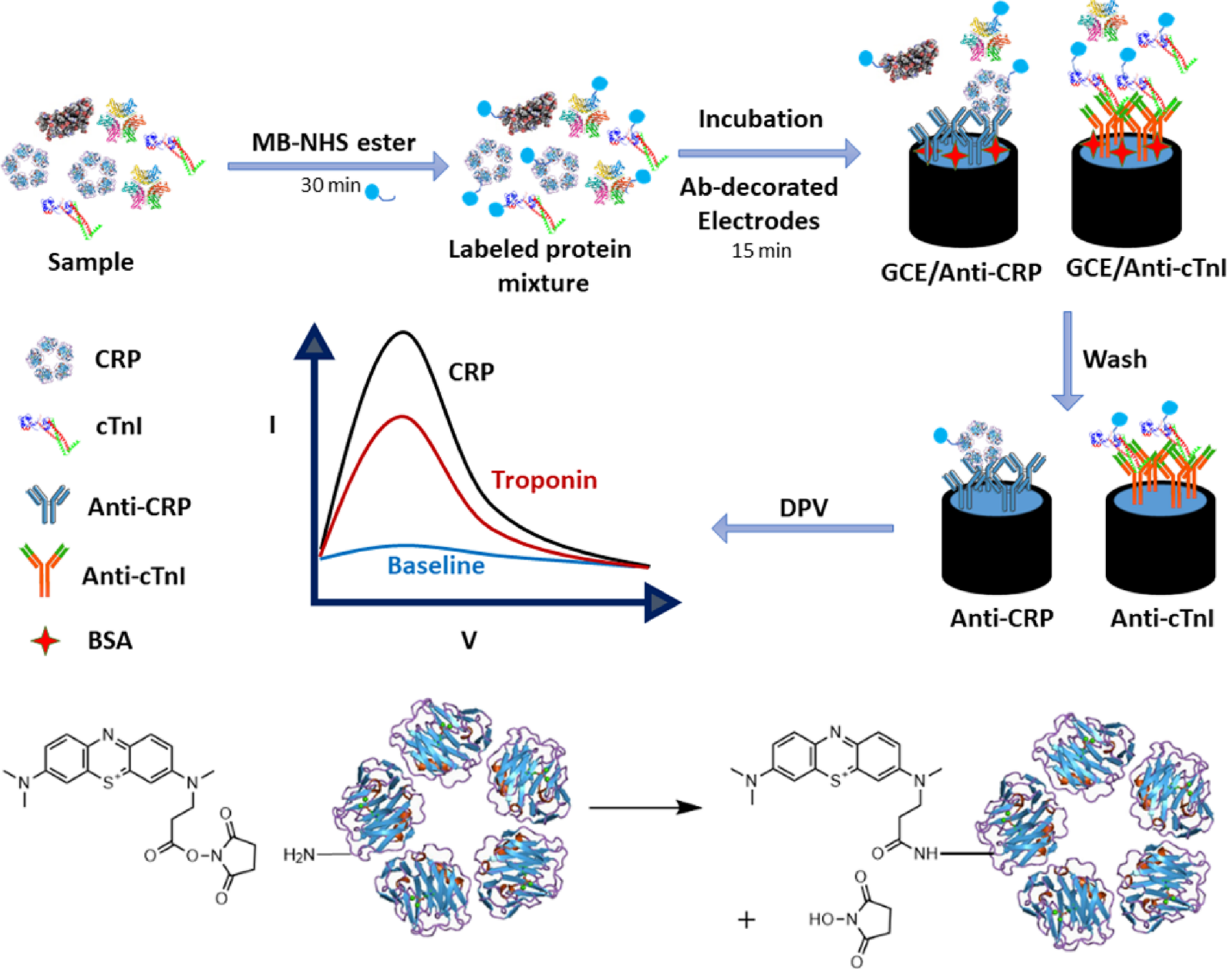
Schematic Depiction of Electrochemical Quantification of Shotgun Redox-Labeled Proteins (Top) and the Coupling Reaction of Methylene Blue NHS Ester to the Free Amine Groups on CRP (as an Example Analyte) via Amide Bond Formation (Bottom) Protein samples are nonspecifically tagged and then captured at antibody-coated electrodes. A concentration-dependent increase in a differential pulse voltammetric signal is used to quantify different targets at each electrode.

To omit the potentially laborious (and costly) construction and characterization of sandwich tags (such as redox-tagged antibodies), we herein exploit the Faradaic quantitation of directly redox-tagged targets captured at an antibody-functionalized electrode. We utilize nonspecific succinimidyl ester tagging of proteins in serum prior to immunorecognition-mediated target capture and voltammetric quantification of two important cardiovascular biomarkers: C-reactive protein (CRP)^30^ and cardiac troponin I (cTnI)^31^ (Scheme 1). This is a generalizable methodology that supports biomarker assaying at low detection limits (<1 pg/mL) from real biological samples. We further demonstrate the integration of this methodology into a simple microfluidic mixing format that reduces assay time to minutes in a semiautomated format. This strategy lies at the interface between labeled (probe-tagged targets) and label-free techniques (single-step immunorecognition with no secondary labeling event).

## Materials and Methods

### Materials and Equipment

If not otherwise stated, all chemicals used were purchased from Sigma-Aldrich. Methylene blue-NHS ester was purchased from Glen Research and used as provided. Nafion 117 4% solution in a mixture of lower aliphatic alcohols and water was purchased from Sigma-Aldrich and used as obtained. Goat anti-human CRP polyclonal antibodies (1707-0189G) and native human CRP (1707-2029) were purchased from BioRad. Anti-cardiac troponin I antibody (M155) (ab10237) and recombinant human cardiac troponin I (cTnI) protein (ab207624) were from Abcam. Water used throughout buffer preparation was ultrapurified with a resistivity of 18.2 MΩ cm (Milli-Q Direct/Merck Millipore). Fibrinogen, bovine serum albumin (BSA), human serum albumin (HSA), fetal bovine serum (FBS), and human serum were purchased from Sigma-Aldrich. All electrochemical measurements were carried out with a three-electrode setup using a PalmSens 4 potentiostat powered by PSTrace 5.8. The SPR measurements were performed on a Reichert DC7200 using integrated SPRAutolink software. 3D-printed microfluidic chips were designed using Fusion 360 software (Autodesk) and manufactured using an ELEGOO Mars UV-photocuring LCD printer with an ELEGOO translucent resin. Leak-free Ag/AgCl reference (LF-2-100) electrodes were purchased from Alvatek. Glassy carbon disk electrodes (GCE) with diameters of 3.0 mm were from CHI. Gold disk electrodes with diameters of 2.0 mm were from CHI. The outer diameter for both electrodes was 6.4 mm.

### Antibody Immobilization

Glassy carbon electrodes were polished sequentially with 1.0, 0.3, and 0.05 μm alumina, sonicated for 30 s in 1:1 H_2_O:ethanol, and cycled in 0.5 M potassium hydroxide between 0.70 and 1.70 V vs a leak-free Ag/AgCl reference electrode at 100 mV/s for 20 cycles. They were then incubated for 16 h with 100 μg/mL of Ab at 4 °C. EIS and DPV of antibody-decorated electrodes were measured in 5 mM [Fe(CN)_6_]^4–/3-^ to confirm successful surface functionalization (Figure S11). Electrodes decorated with antibodies were washed with PBS buffer and incubated in 1 M ethanolamine in PBS for 1 h and subsequently with 1% FBS in PBS for 15 min to reduce nonspecific protein binding before measurements.

### Solution-Phase Redox Tagging

Fifty microliters of freshly prepared methylene blue–NHS ester solution (initially at 20 μg/mL but diluted to 10 μg/mL when mixed with the sample) in DMSO was immediately mixed with 50 μL of the protein sample aliquoted in 100 mM MES buffer at pH 6.0, 1% FBS in 100 mM MES, or 1% human serum (HS) in 100 mM MES. The mixture was vortexed and incubated for 30 min without stirring. Twenty microliters of a mixed solution of 1 M ethanolamine and 1 M hydroxylamine were then added to the reaction mixture and incubated for 5 min to quench any unreacted MB-NHS ester and prevent nonspecific binding of free esters to electrode-confined antibodies. The concentration of MB-NHS (10 μg/mL equivalent to 19 μM in the test solution) was chosen in accordance with the manufacturer’s protocol suggesting a 5-fold excess of NHS ester over total protein. Total serum protein concentration was estimated at 50 mg/mL in human serum (4 μM in test solution).

Prepared electrodes were incubated with 15 μL of the labeled protein samples for 15 min, washed with PBS, and immersed in degassed 0.1 M KCl (N_2_-purged for 30 min), and then DPV curves recorded between −0.60 and 0.00 V vs an Ag/AgCl reference electrode with platinum (Pt) counter electrodes. Electrochemical measurements were performed using a PalmSens4 potentiostat, and DPV recorded at a 100 mV/s scan rate with 50 mV pulses in 0.01 s. Peak heights were calculated after subtracting the background current at ≈−0.50 V (vs Ag/AgCl), a potential consistent with previous literature on methylene blue–NHS conjugates.^32−34^ Current densities were calculated relative to the geometric electrode surface area (3.0 mm diameter).

### Specificity Studies

High concentrations of common interfering proteins (2 mg/mL BSA, 2 mg/mL HSA, and 2 mg/mL fibrinogen) were labeled with MB-NHS as described above, and the tagged solutions were then incubated with antibody-modified electrodes. DPV signals were recorded and used to compare specific target protein responses (CRP or cTnI). The cross-reactivities of both target proteins toward their antibodies were also studied by incubating anti-CRP-modified electrodes with a high concentration of cTnI (100 ng/mL) and incubating anti-cTnI electrodes with a high concentration of CRP (200 ng/mL) and comparing responses to those generated by respective specific targets.

### Design and Construction of the Microfluidic Chip

The microfluidic chip (Figure 4 and Figure S3) was designed using Fusion360 software and subsequently printed using an ELEGOO UV-curing 3D printer. A reference electrode was prepared by electrolysis in 0.1 M HCl at a plain silver wire (0.25 mm diameter). Subsequently, the so-formed reference electrode was coated with Nafion 117 by eight repeated cycles of incubation in 4% Nafion solution and air-drying. This and a platinum wire (0.20 mm diameter) counter electrode were then inserted into the microfluidic cell and fixed via a commercial adhesive epoxy resin (Araldite).

### Microfluidic Assay and Measurement

An antibody-coated electrode was introduced into the microfluidic chip and fitted tightly. Inlets of the microfluidic cell were connected to a Harvard Apparatus Standard Infuse/Withdraw PHD 2200 syringe pump. Before each measurement, the microfluidic cell was washed with DI water and dried with nitrogen, and then the two loading chambers (Figure 4) separately filled with 25 μL of 20 μg/mL MB-NHS in DMSO and 25 μL of protein solution. The loading apertures were then closed using Kapton tape and 0.1 M KCl pumped through both inlets of the cell with a flow rate of 10 μL/min for 5 min, allowing the two solutions to actively mix inside the flow channel. When the electrode chamber was filled, the flow was stopped for 10 min to incubate the electrode with the tagged protein. Subsequently, the cell was washed with 0.1 M KCl at a flow rate of 100 μL/min through each inlet for 2 min. During this, the flow cell was held with a slight positive angle toward the outlet end to prevent the formation of air bubbles. DPV curves were then recorded in 0.1 M KCl between −0.6 and 0.0 V vs the Ag/AgCl/Nafion reference electrode with a Pt counter electrode at a 100 mV/s scan rate and 50 mV 0.01 s pulses.

### Microfluidic Spike Recovery

For the spike recovery experiments in the microfluidic setup, selected known concentrations of both proteins were first analyzed to correct for interelectrode variation. Electrodes were then exposed to spiked human serum samples, and the measured DPV response used to estimate target protein concentration. Recovery was calculated as the ratio of resolved concentrations to the spiked concentration using linear semi-log fitting (see insets in Figure 5).

## Results and Discussion

Optimal assay conditions were initially determined by surveying the generated Faradaic response to targets as a function of buffer pH and tag (MB-NHS) concentration being optimal in MES buffer at pH 6.0 (Figure S1), an observation broadly consistent with known succinimidyl ester coupling efficiencies.^35^ Nonspecific labeling was further confirmed using an SDS-PAGE analysis, where MB-tagged proteins showed a characteristic fluorescent signal (Figure S2). Simultaneously, a series of MB-NHS concentrations were tested to achieve a maximum signal-to-noise ratio, with 10 μg/mL (19 μM final concentration, 5-fold higher than the estimated total serum protein as rationalized in the Methods section) being optimal and generating a dynamic range spanning over 5 orders of magnitude (Supplementary Information).

**Figure 1:**
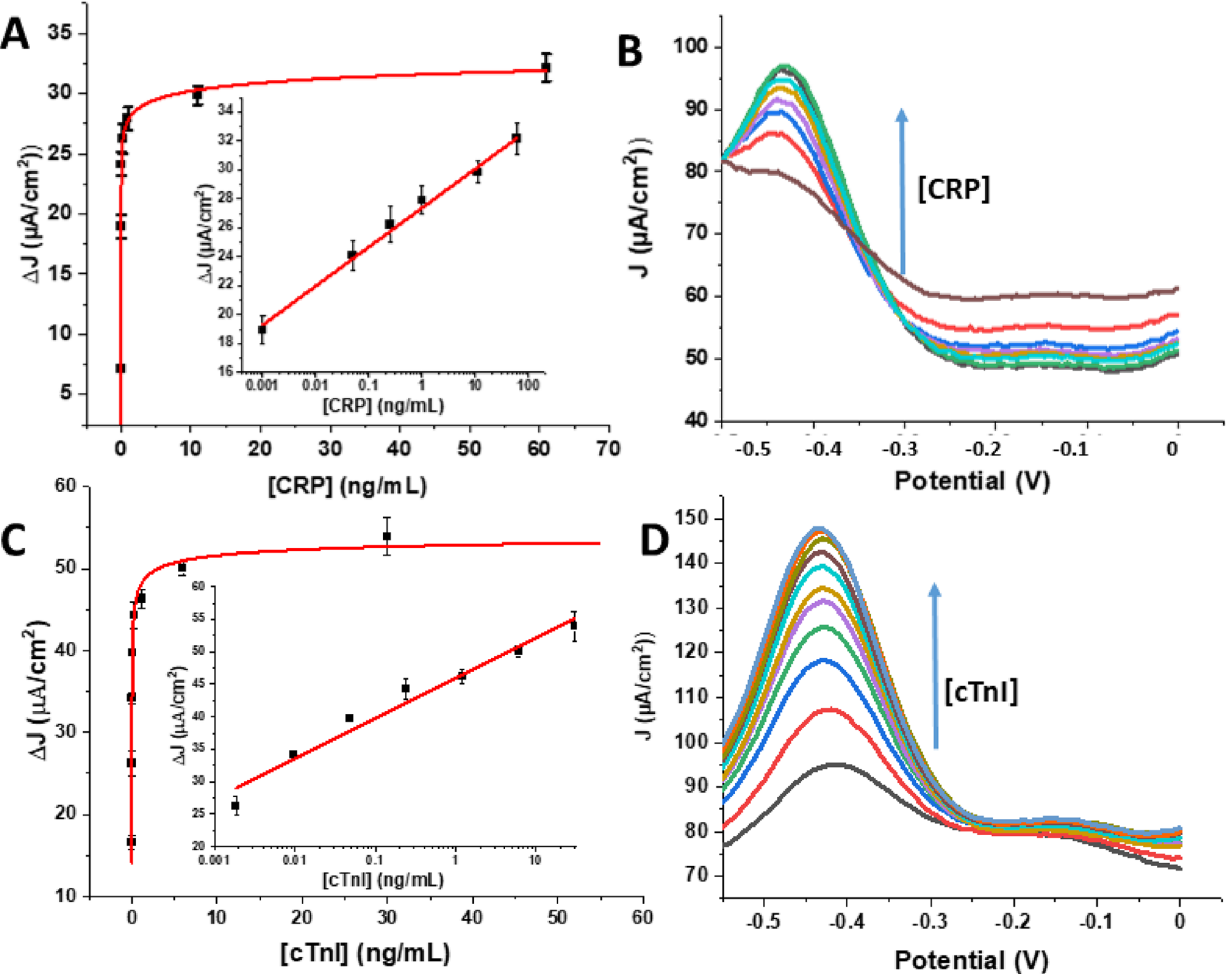
Langmuir–Freundlich fits of (A) CRP spiked in 1% human serum in MES buffer and (C) cTnI spiked in 1% human serum in MES buffer. Representative DPV peaks as function of (B) increasing CRP connection on anti-CRP-decorated GCE sensors and (D) cTnI at anti-cTnI sensors. Bottom DPV voltammograms in (B) and (D) are the background signals from incubation with unspiked 1% HS. Error bars represent standard deviations between three independent measurements at three different electrodes. Insets in (A) and (C) are associated linear semi-log correlation trends with correlation coefficients (*R*^2^) of 0.99 and 0.97, respectively.

These target protein-labeling protocols showed no significant effect on the affinity of immobilized antibodies to their respective target as indicated by SPR analyses (Figure S3); there was no statistically significant difference in the observed dissociation constants (*K*_D_) for MB-labeled CRP (9.5 × 10^–10^ M) and free CRP (2.8 × 10^–10^ M) comparable to the values obtained from electrochemical Langmuir–Freundlich fitting (5.0 nM) as an approximation of the thermodynamic properties of the system.^36^ Under these optimized conditions, assays were then performed by spiking specific concentrations of either CRP or cTnI into dilute human serum. Samples were tagged by incubating with MB-NHS for 30 min after vortexing for 10 s prior to reaction quenching through the addition of ethanolamine and hydroxylamine. Labeled samples were then incubated with antibody-modified electrodes for 15 min prior to DPV sweeps in 0.1 M aqueous KCl solution. Good semi-log correlations were observed between specific CRP/cTnI concentrations and DPV peak currents (−0.4 V) with detection limits (LOD) of 1 pg/mL (CRP) and 0.3 pg/mL (cTnI) and dynamic ranges spanning from 1 pg/mL to 100 ng/mL (CRP) and from 0.6 pg/mL to 20 ng/mL for cTnI (Figure 1). DPV signals were saturated thereafter with nonsignificant increments in current densities, indicating the signal at plateau (Figure S7).

**Figure 2:**
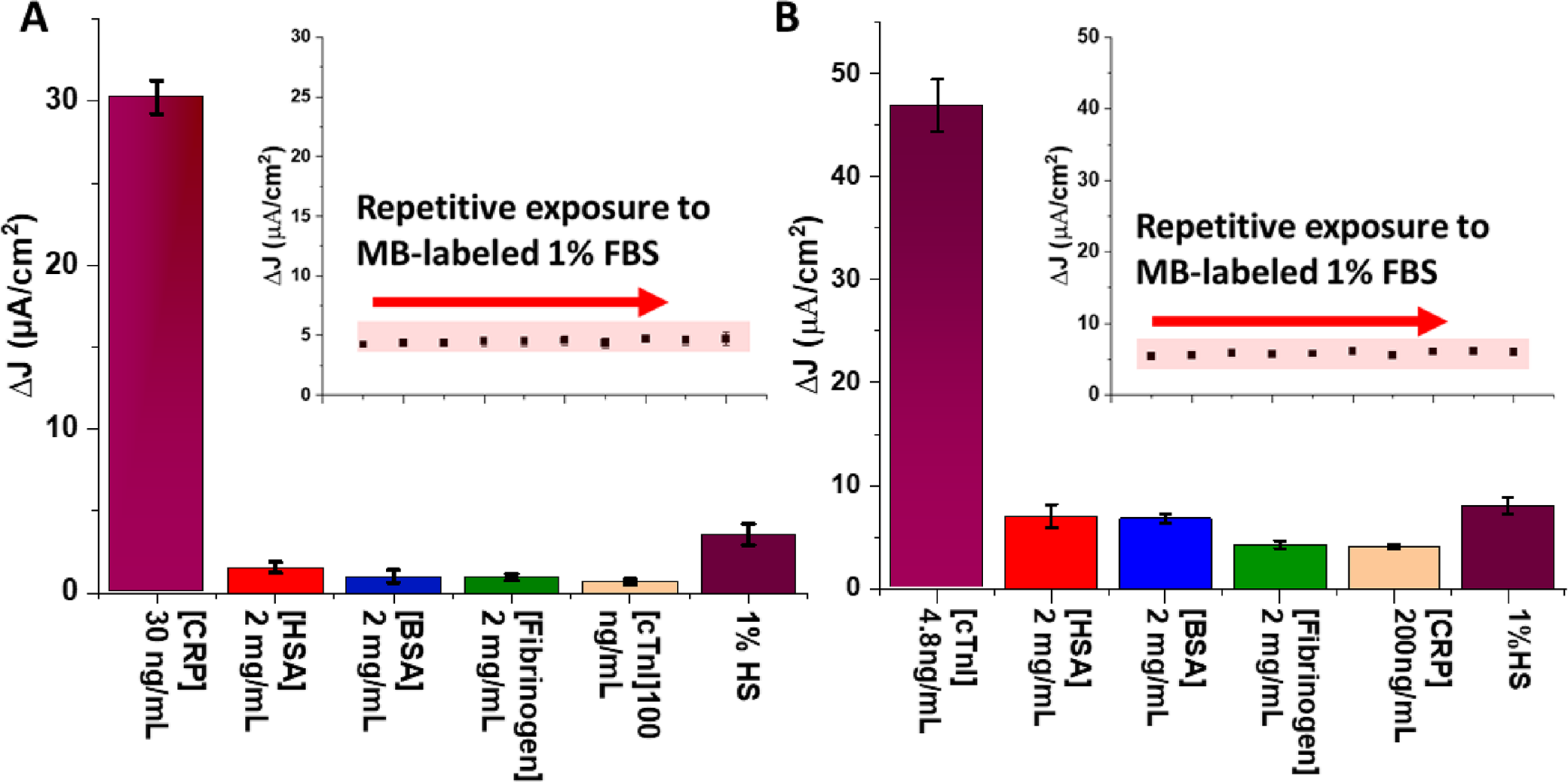
Electrochemical specificity analysis for (A) anti-CRP-modified electrodes and (B) anti-cTnI-modified electrodes in offline single protein analyses. The response (current density) to a large excess of interfering species is <10% of the target-specific signal at anti-CRP interfaces and <20% across both surfaces with all interferents, indicative of good specificity and low cross-reactivity (without the need for complex surface chemistry). Insets show the response of the anti-CRP-modified electrode (A) and the anti-cTnI-modified electrode (B) upon repetitive exposure to MB-labeled 1% fetal bovine serum. Such analyses confirm that the signal is target-specific with minimal (consistently less than the assay LOD (blank + 3 × SD)) contribution from interfering species. Error bars represent standard deviation from three independent measurements at three different electrodes. Note that specificity is yet further improved under flow (Figure S8).

The ability to robustly differentiate between specific targets and nonspecific binding was assessed by measuring the voltammetric response upon challenging sensors with (tagged) common proteins expressed in human serum, human serum albumin (HSA), fibrinogen, and bovine serum albumin (BSA). The antibody cross-reactivity was additionally examined by testing responses to high concentrations of CRP at anti-cTnI-modified electrodes and vice versa. All observations are indicative of high levels of selectivity and in agreement with analogous SPR analyses (Figure 2 and Figure S4). Antibody-free electrodes (BSA-coated) showed signals less than 5% (less than the assay LOD (blank + 3 × SD)) of the specific signals observed for any of the studied proteins on their respective antibody-modified electrodes (Figure S5). Additionally, repetitive exposure of anti-CRP- or anti-cTnI-modified electrodes to MB-labeled 1% FBS generates no change in background signal (remaining <5% of the specific signal), further confirming that the Faradaic DPV signal is only dependent on the specific recruitment of target analytes with little contribution from nonspecific protein adsorption on the electrode surface (Figure 2, insets).

**Figure 3:**
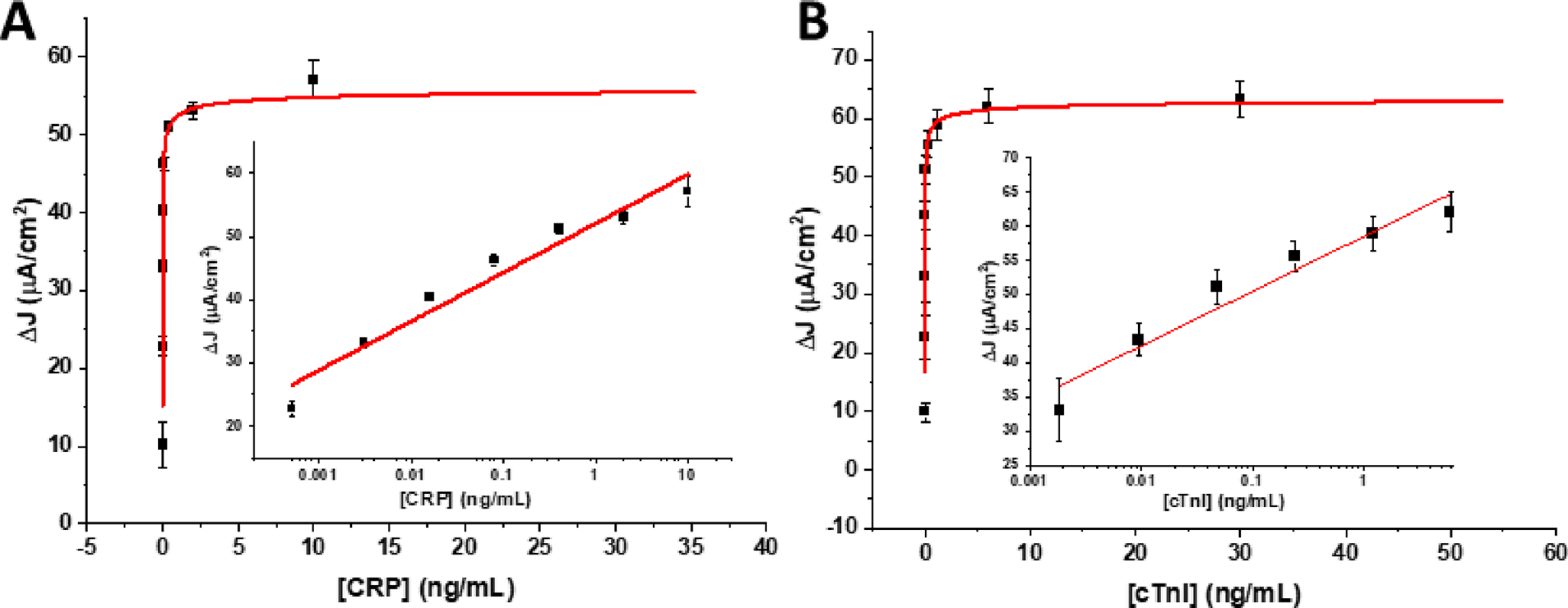
Langmuir–Freundlich fits from offline multiplexed protein assays as detected from mixed samples of both proteins at a CRP-responsive sensor (A) and a cTnI-responsive sensor in dilute serum (B). Respective LODs are 1.0 pg/mL for CRP and 0.6 pg/mL for cTnI. Insets depict associated linear semi-log correlation plots with correlation coefficients (*R*^2^) of 0.95 and 0.94 for CRP and cTn1, respectively. Error bars represent standard deviations between three independent measurements on three different electrodes.

In an assessment of offline multiplexing, MB-labeled mixtures of CRP and cTnI (spiked in 1% human serum) were assessed at two electrodes (one decorated with anti-CRP and the other decorated with anti-cTnI). The resulting responses (Figure 3) are very comparable to those observed with single-protein spikes, again indicating low levels of interface and antibody cross-reactivity.

**Figure 4:**
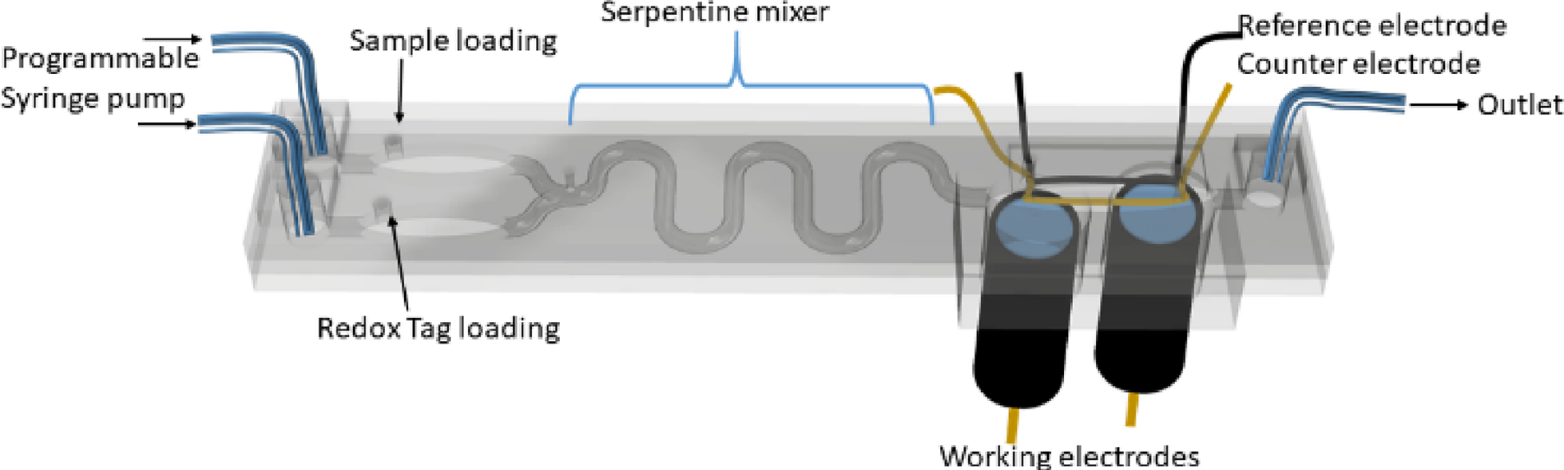
Schematic representation of the microfluidic chip used for sample handling and electrochemical detection. The chip encompasses an electrode compartment that houses two working disc electrodes (6.4 mm outer diameter) with reference Ag/AgCl and counter platinum electrodes. The electrode compartment is connected to a mixing serpentine channel designed to mix samples and reagents pumped from their respective chambers. Forty microliters of sample and reagent are loaded into these chambers through an injection port. This is then closed using adhesive tape and connected, using flexible tubing, to a syringe pump programmed for 5 min of flow through the serpentine channel, 10 min of incubation, and 1 min of washing.

A microfluidic Y-shaped serpentine mixer was then designed and utilized to improve sample/reagent mixing and assay time within a closed low-volume chamber directly accessible by a syringe pump. Initial assessments were carried out with CRP alone (Figures S6 and S7) and then extended to the simultaneous detection of both markers spiked into human serum. The inline integration of two sensor electrodes enables dual-marker quantification (sample-to-answer) within 15 min without the need for reagent quenching (Figure 4); the sample and the MB tag are loaded by pipetting into their respective chambers prior to mixing by pumping at a preoptimized flow rate (20 μL/min). The mixture is then incubated at the electrode chamber for 10 min followed by washing in running 0.1 M KCl solution at 100 μL/min for 60 s and simultaneous DPV analysis at both working electrodes. Under such conditions, assay specificity is further improved from that observed in the offline/static solution assay as indicated by the notably lower response to interfering species (<10% for both anti-CRP- and anti-cTnI-modified electrodes; Figure S8). This improved performance within the confines of a flowing microfluidic platform probably arises from a mixture of improved mass transport and a greater suppression of nonspecific adsorption as afforded by the enhanced mixing and washing.^37,38^

**Figure 5:**
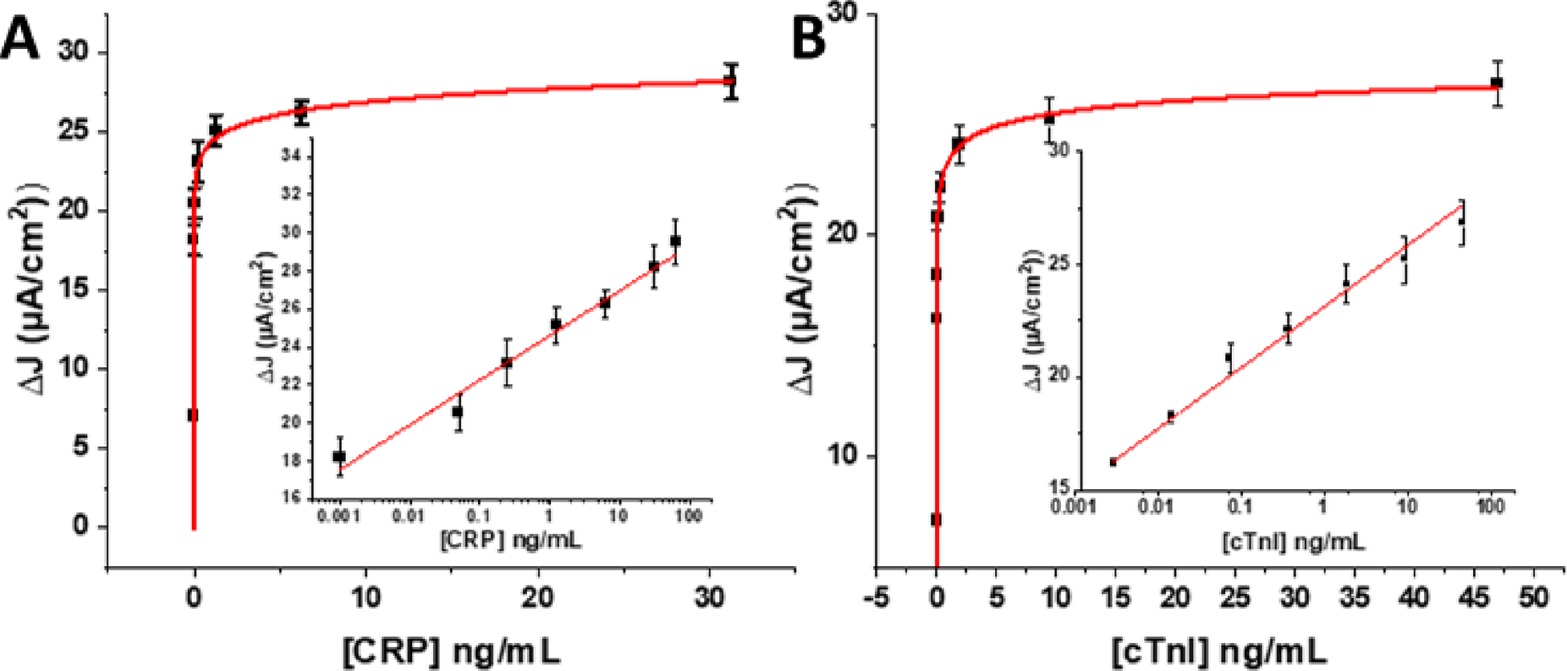
Representative Langmuir–Freundlich fits from online multiplexed assays for (A) CRP and (B) cTnI both spiked in 1% human serum in 0.1 M MES buffer. Insets represents linear semi-log correlation with correlation coefficients (*R*^2^) of 0.978 for CRP and 0.993 for cTnI. Error bars represent standard deviations between three independent measurements on three different electrodes.

The online microfluidic protein configuration supports very high levels of assay reproducibility and accuracy with linear dose–response trends (Figure 5 and Figure S9) across 1.0–100 ng/mL CRP and 3–62.5 ng/mL cTnI dynamic ranges. Standard deviations between three independent measurements on three different sensor electrodes per protein target were less than 10%, indicating very good levels of reproducibility. An analysis of assay accuracy, as tested by spiked recovery experiments in serum, demonstrated high diagnostic performance (Tables 1 and 2). To confirm that these peaks were Ab-Ag interaction-specific, bare electrodes were exposed to free and MB-tagged proteins. Electrodes showed no Faradaic responses to free proteins, while large signals were observed for all MB-tagged proteins, confirming a robust tagging protocol (Figure S12).

**Table 1:** Spike Recovery of CRP in 1% Human Serum

added CRP conc. (pg/mL)	recovered conc. (pg/mL)	percent recovery
261	308	118%
661	664	100%
761	762	100%
1761	1626	92%

**Table 2:** Spike Recovery of cTnI in 1% Human Serum

added cTnI conc. (pg/mL)	recovered conc. (pg/mL)	percent recovery
110	118	107%
466	485	104%
1152	1095	95%
1728	1784	103%

## Patient Sample Analysis

**Figure 6:**
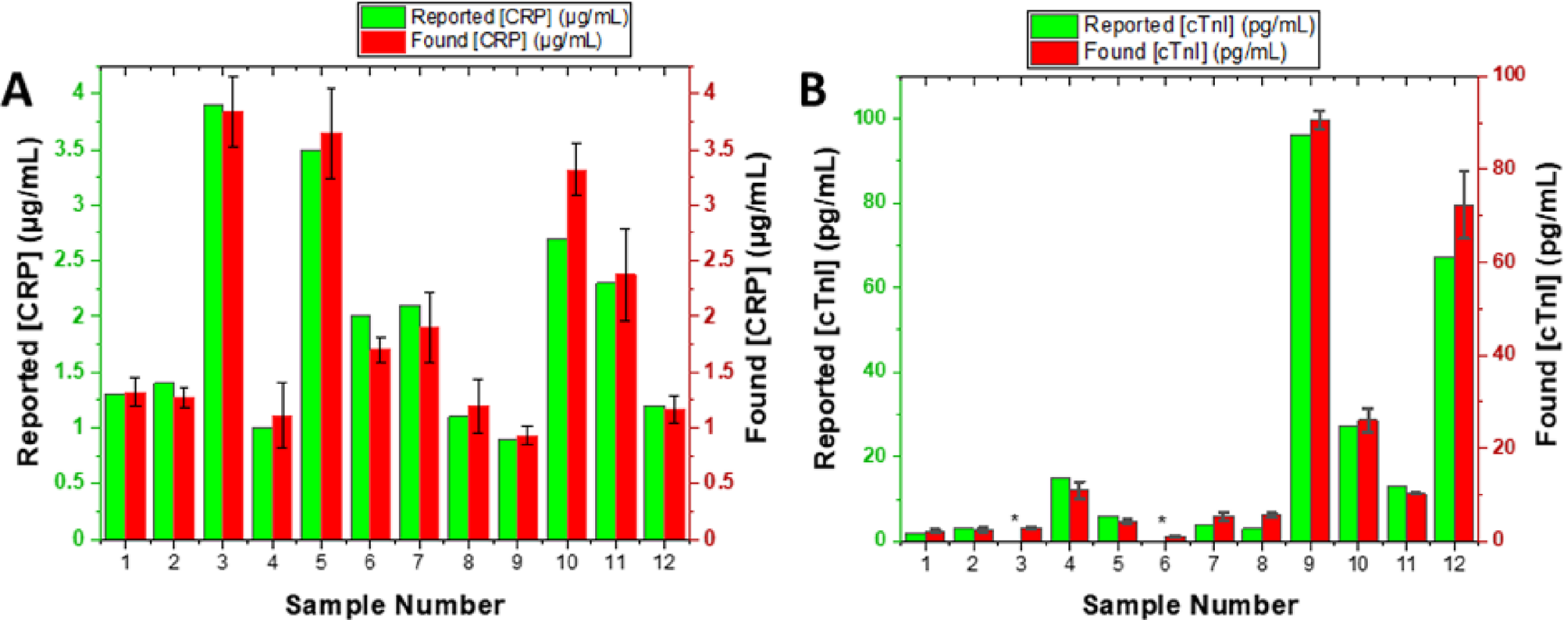
Blind comparison of CRP concentrations (A) and cTnI concentrations (B) estimated using the shotgun protein-tagging assay against a reference immunoassay for 12 patient samples (asterisks represent samples where cTnI levels were lower than the immunoassay LOD, i.e., <2 pg/mL). Error bars represent standard deviations from two independent measurements on two different electrodes.

The simultaneous detection of CRP and cTnI can greatly enhance early detection, reducing mortality and improving treatment outcome for cardiovascular disease (CVD).^39−41^ The analyses above, with detection limits as low as 0.6 pg/mL in serum and dynamic ranges spanning over 6 orders of magnitude, compare favorably with most recent electrochemical platforms for sensing of CRP and cTnI,^41^ requiring only 25 μL/assay and 15 min of total analytical time (sample dilution to readout; well below recognized guidelines recommending an analysis within 1 h of patient admittance, a target that challenges the sensitivity of current clinically available methods).^42^ To demonstrate the clinical applicability of the proposed assay, randomized patient samples were analyzed and estimated concentrations of CRP and cTnI were compared to immunoassay results from an Abbott Architect system (Figure 6). Both CRP (Figure 6A) and cTnI (Figure 6B) analyses show a good correlation; correlation coefficient and slopes were near unity and intercept close to zero (Figure S10).

## Conclusions

We have presented a facile single-step assay for the electrochemical detection of two common cardiac biomarker proteins, CRP and cTnI, as model protein targets. The methodology omits any complex or cost-intensive steps involved in developing specifics (e.g., antibody-based labels) while providing a signal turn-on assay and high specificity format that combines the advantages of label-free and sandwich-type assays. It can readily be applied to different target proteins within a sample, making it a very accessible and cost-effective means of multimarker quantification. The assays achieve very low detection limits (<1 pg/mL) and dynamic ranges spanning over 5–6 orders. Minimum user intervention and >15 min assay times are achieved via integration within a semiautomated microfluidic format. Clinical applicability has further been demonstrated with a blind patient sample recovery analysis, which showed an excellent correlation to standard immunoassay results for both markers with a notable ability to resolve ultralow concentrations of cTnI. The proposed methodology is of general applicability, operates well in complex biological samples, and is of low cost (below USD 5/sample consumable cost compared to USD 35/sample for conventional ELISA).^43^ Microfluidic integration offers a potentially fully automated and highly scalable application to point-of-care use with minimum user training. We believe this study to be of value in both further extending the realm of electrochemical biosensing methodologies and contributing to the challenge of finding smart solutions for societal healthcare needs.

## Supporting Information Available

pH and concentration optimization; SDS-PAGE analysis of MB-tagged protein vs free protein; SPR affinity analysis of anti-CRP toward free and MB-labeled CRP; assay specificity; responses of antibody-free electrodes toward labeled proteins; microfluidic design for single-protein quantitation; online microfluidic CRP quantitation; online assay specificity; representative DPV peaks from multiplexed protein analysis; assay validation; patient sample analyses; electrochemical study of antibody immobilization (PDF)
