# Supplementary material for: A Quantification of Target Protein Biomarkers in Complex Media by Faradaic Shotgun Tagging
Source: Anal Chem. 2022 Jan 27;94(5):2375–82. doi: 10.1021/acs.analchem.1c03695 (PMC9082491; doi:10.1021/acs.analchem.1c03695)
Supplement: Supplementary file 1 — ac1c03695_si_001.pdf [file ac1c03695_si_001.pdf]

Supporting information

A quantification of target protein biomarkers in complex media by faradaic shotgun tagging

Mohamed Sharafeldin;[1*] Felix Fleschhut;[1*] Timothy James;[2] Jason J. Davis[1]

[1] Department of Chemistry, University of Oxford, South Parks Road, Oxford OX1 3QZ, UK

[2] Department of Clinical Biochemistry, Oxford University Hospitals NHS Trust, Oxford, OX3 9DU, U.K.

[*]Authors contributed equally to the manuscript

Table of Contents

pH and concentration optimization 2

SDS-page analysis of MB-tagged protein Vs free protein 3

SPR affinity analysis of Anti-CRP towards free and MB-Labelled CRP 4

Assay specificity study 5

Reponses of antibody-free electrodes towards labelled proteins 6

Microfluidic design for single protein quantitation 7

Online microfluidic CRP quantitation 8

Online assay specificity study 9

Representative DPV peaks from on-line multiplexed protein analysis 10

Assay validation 11

Patient sample analyses 12

Electrochemical study of antibody immobilization 13

Electrochemical study of free and MB-Tagged proteins adsorbed on bare GCE 14

pH and concentration optimization

To assess the effect of pH on the efficiency of coupling between the MB-NHS and proteins, the assay performance was tested using different pH buffers for the protein solution. These were pH 5.0, 6.0, 7.0 and 8.0 in acetate, MES and phosphate (pH 8.0, pH 9.0) buffers respectively. The results shown in figure S1 demonstrate the change in response as function of CRP concentration at studied buffers. The coupling in 100 mM MES buffer at pH 6.0 showed highest signal to noise ratio and a larger dynamic range. This was accordingly applied thereafter as the dilution medium.

Two different concentrations of methylene blue NHS were tested to investigate the effect on its concentration on the electrochemical behaviour of the assay. At high MB-NHS concentration (100 µg/mL) the detection limit was 500 pg/ml largely due to the high background signal. Reducing MB-NHS conc. to 10 µg/mL resulted in a much lower background decreasing the assay LOD down to 1 pg/mL. This ensured a low assay S/N ratio at the optimized experimental and electrochemical parameters reducing the background relevant to the amount of non-specifically adsorbed proteins.

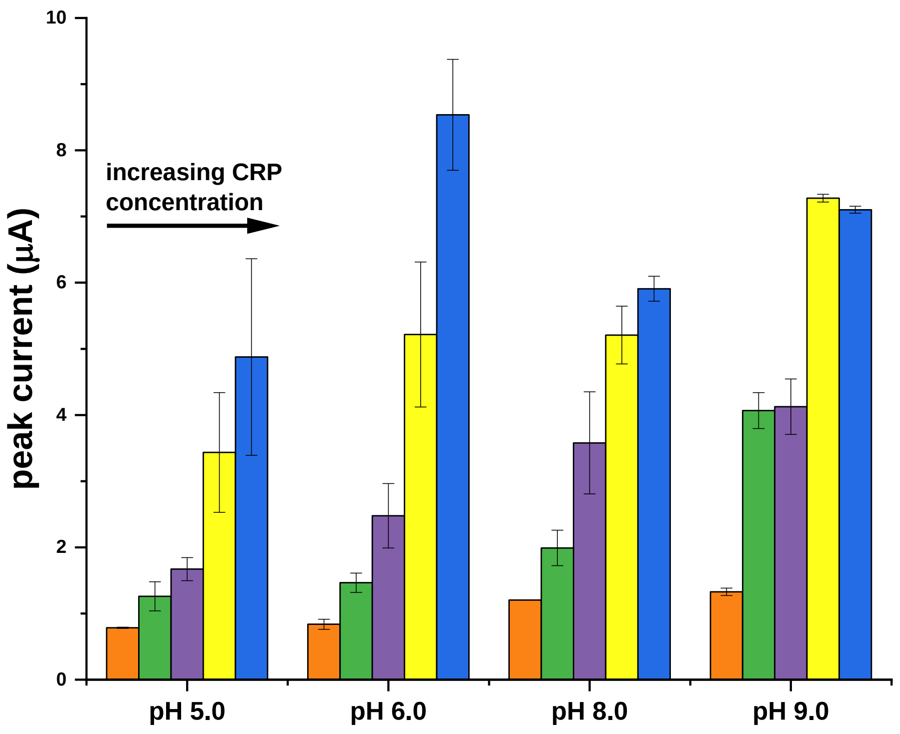

Figure S1: Optimization of pH buffer labelling conditions. Columns show average of response over two independent measurements with increasing CRP concentration for protein solutions buffered at different pH values. Buffers used were 100 mM Acetate buffer at pH 5.0, 100mM MES buffer at pH 6.0 and 100mM Potassium phosphate buffer at pH 8.0 and pH 9.0.

SDS-page analysis of MB-tagged protein Vs free protein

The successful covalent tagging of protein with the MB-NHS label was confirmed by in-gel fluorescence. The samples were mixed with 4xLaemmli Buffer, boiled for 5min at 95 °C and 15 µg protein loaded onto 4-12% Criterion® XT Bis-Tris protein gel, 26 well (Biorad©, #3450125) in 1x MES buffer (170 V, 55 min). Samples were prepared by labelling 1mg/mL BSA in MES buffer according to the standard preparation as for the assay. Methylene blue shows fluorescence upon absorption of light around a wavelength of 665 nm and emits light with a peak at around 686 nm. Therefore, the labelled BSA shows fluorescence (imaged on a Li-Cor Oddysey© DLx) when illuminated in the gel, while the fluorescence is absent for the native BSA. On the contrary Coomassie staining labels both proteins native and labelled at the expected mass of around 66kDa.

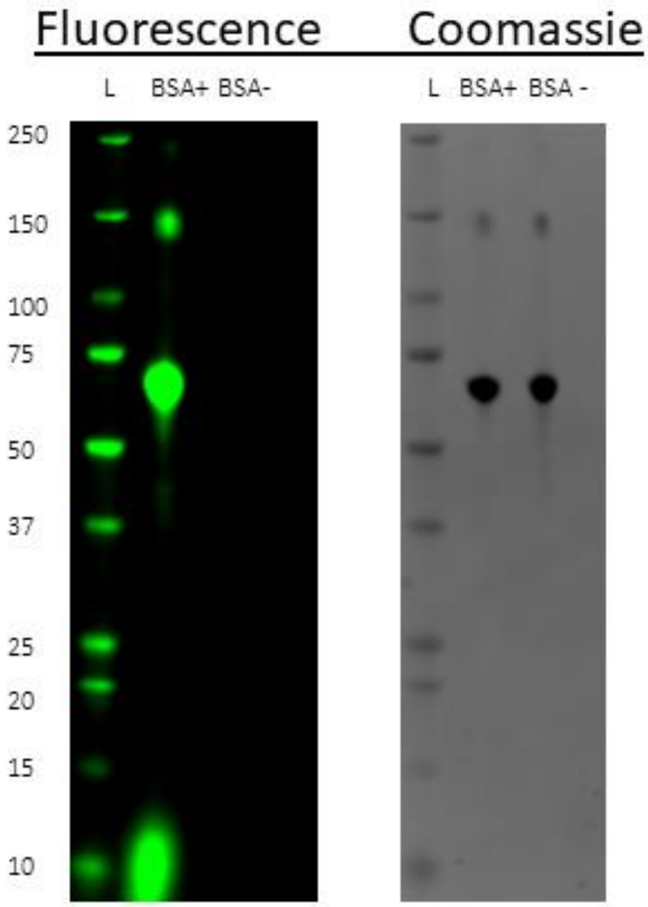

Figure S2: In-gel fluorescence and Coomassie images obtained from gel electrophoresis of native and MB-labelled BSA protein. Both pictures show the same gel with in-gel fluorescence and Coomassie stain with a fragment ladder, the labelled BSA and the native BSA from left to right, respectively.

SPR affinity analysis of Anti-CRP towards free and MB-Labelled CRP

Affinities of anti-CRP modified SPR chip towards free and MB-labelled CRP were assessed by injecting a series of 5 concentrations of free CRP, or MB-Labelled CRP on a Biacore© X-100 SPR. The cumulative capture of CRP onto the SPR chip was fitted into a 1:1 Langmuir binding model to estimate the dissociation and association rate constants using Biacore© evaluation software. The apparent dissociation constant (K_D) was calculated as the ratio between the dissociation rate constants to association rate constants (k_d/k_a). Calculated (KD) for free and MB-labelled CRP showed no significant difference with that of free CRP at 2.8 X 10^{-10} M compared to 9.5 X 10^{-10} M for MB-labelled CRP.

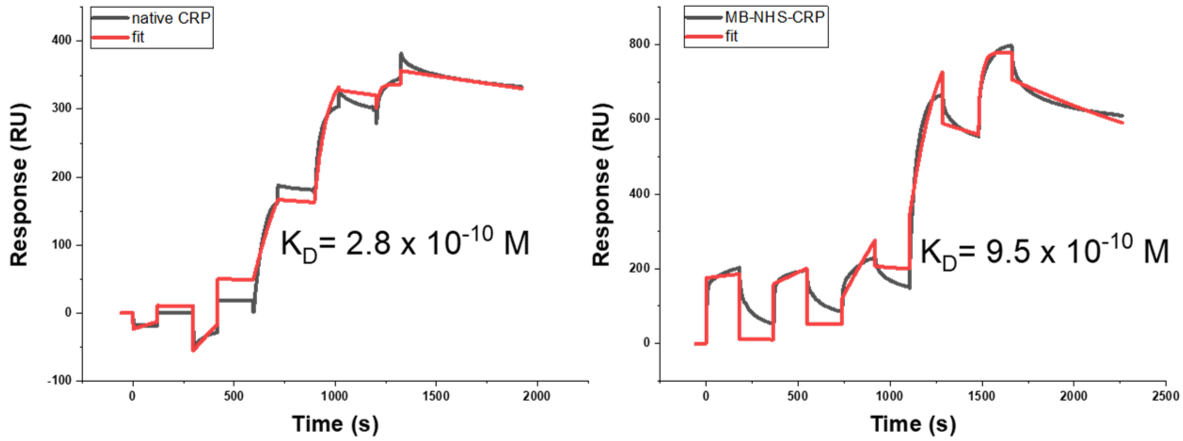

Figure S3: SPR affinity measurements of native CRP (left) and MB-tagged CRP (right) measured on a gold surface with physisorbed CRP antibodies. Black lines depict recorded data, red lines show associated fits obtained from BicaoreX100 Evaluation Software.

Assay specificity study

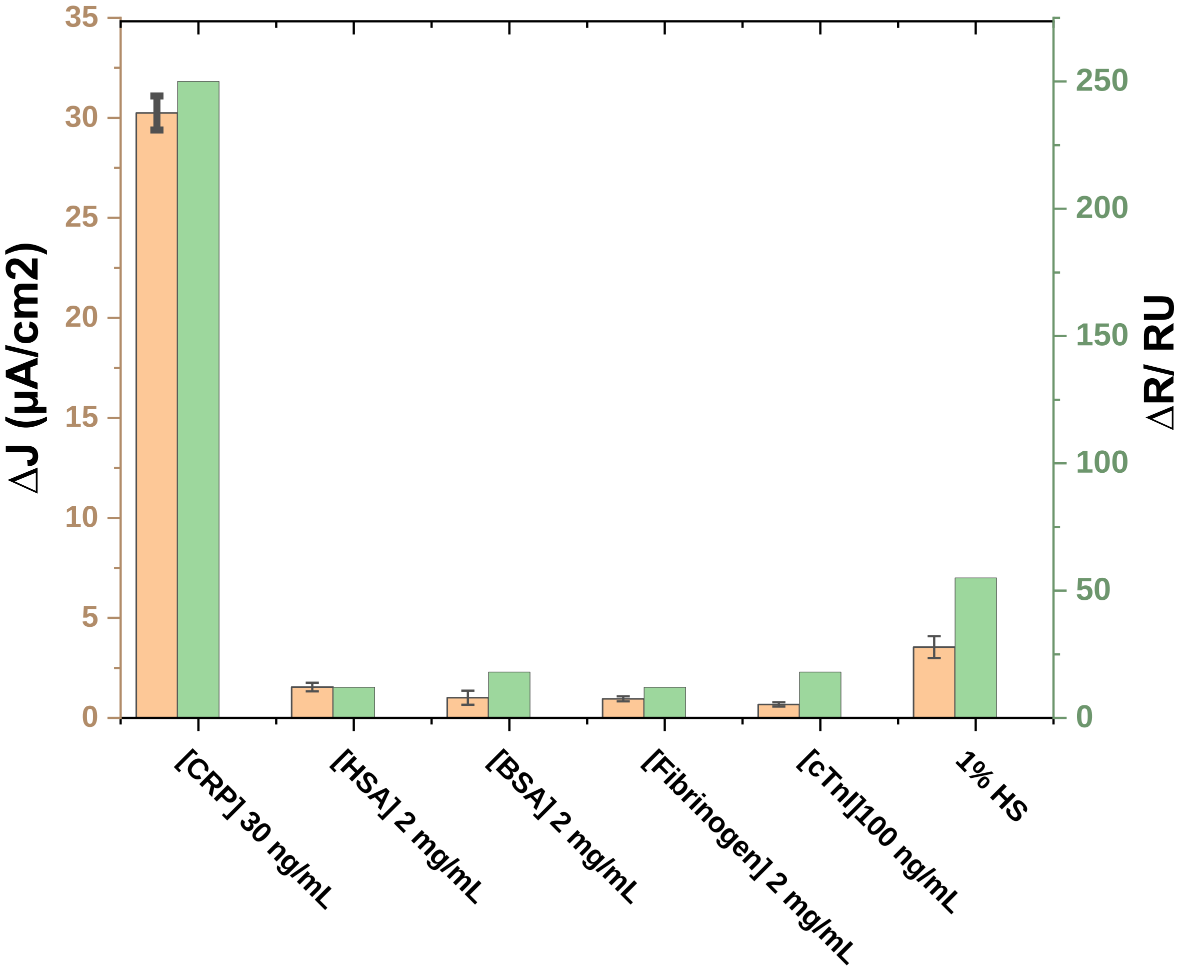

Figure S4. Specificity study for physisorbed anti-CRP interface on GCE electrode and SPR gold chip. Left y-axis represent change in peak current and right y-axis represent change in SPR response units (RU) upon exposing the interface to 30 ng/mL CRP, 2mg/mL human serum albumin, 2 mg/mL bovine serum albumin, 2 mg/mL fibrinogen, 100 ng/mL cTnI, and 1% human serum. All measurements were in MES buffer. The concentrations used represent estimates for protein content of 10 to 100 times diluted human serum.

Reponses of antibody-free electrodes towards labelled proteins

Electrodes decorated with 100 µg/mL BSA (antibody-free interface) were exposed to various concentrations of common proteins labelled with MB-NHS ester including 100 µg/mL BSA, 100 µg/mL HSA, 10 ng/mL CRP, 100 ng/mL CRP, 10 ng/mL cTnI, and 100 ng/mL cTnI. Recorded electrochemical faradic responses were within background noise indicating minimal non-specific binding while confirming that faradaic signals recorded on Ab-modified electrodes are induced by the specific recruitment of target species.

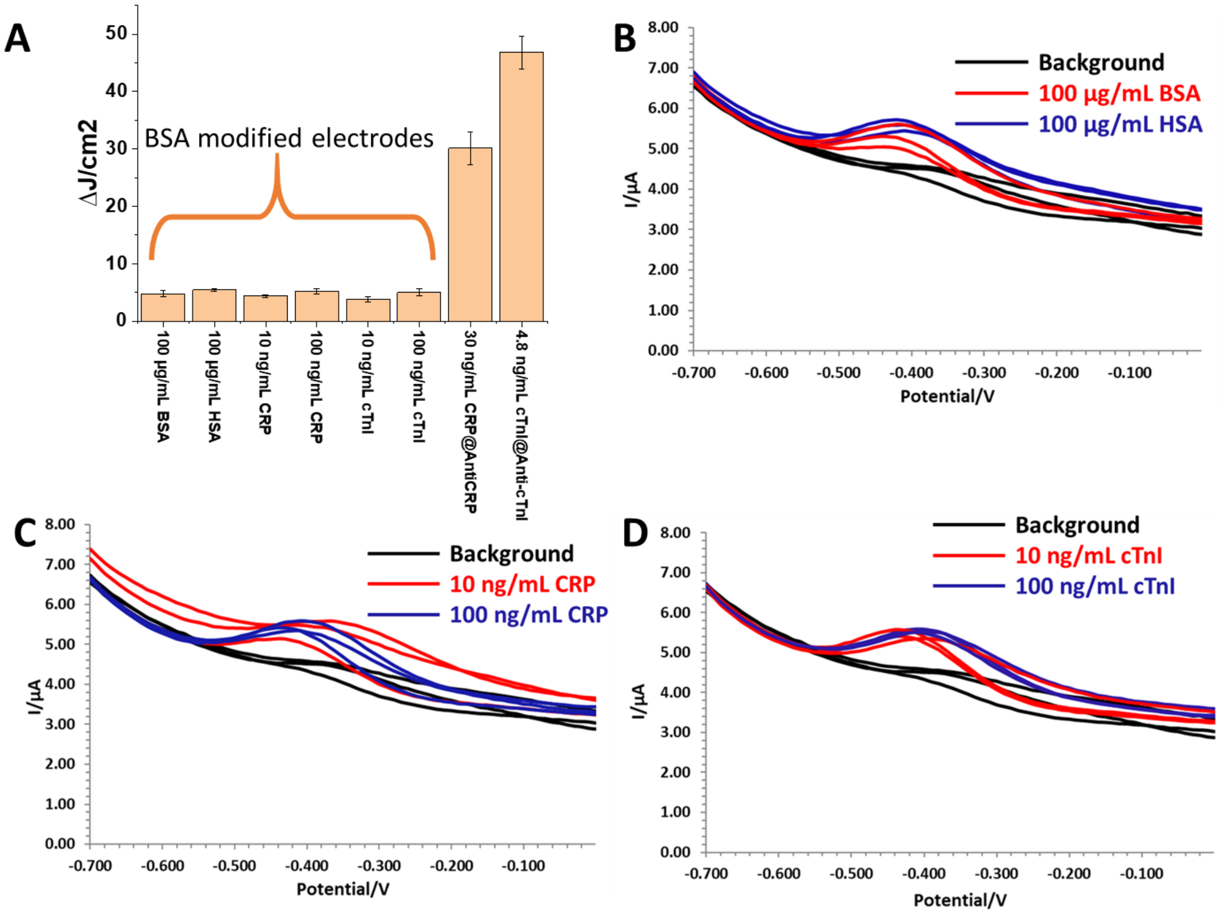

Figure S5: Response of BSA modified electrodes towards MB-labelled proteins. (A) Electrochemical response of BSA modified electrodes towards 100 µg/mL BSA, 100 µg/mL HSA, 10 ng/mL CRP, 100 ng/mL CRP, 10 ng/mL cTnI, and 100 ng/mL cTnI as compared to specific response of anti-CRP modified electrode to 30 ng/mL CRP and anti-cTnI modified electrode against 4.8 ng/mL cTnI. Raw unprocessed voltammograms of BSA electrodes against (B) 100 µg/mL BSA and 100 µg/mL HSA; (C) 10 ng/mL CRP, 100 ng/mL CRP; and (D) 10 ng/mL cTnI, and 100 ng/mL cTnI.

Microfluidic design for single protein quantitation

A microfluidic Y-shaped serpentine mixer was designed and investigated to improve sample/reagent mixing while housing a closed sample and reagent chambers that can be directly driven by a syringe pump. This setup allowed the whole assay to be run from sample-to-answer within 15 minutes without the need for separate mixing or washing procedures. In the proposed design, once reagent and sample are loaded, loading holes were blocked with adhesive tape promising an easy sample loading for a closed microfluidic system. The mixing is induced by pumping both sample and reagent simultaneously at a pre-optimized flow rate (20 µL/min) for efficient mixing. Once the mixture reaches the incubation chamber, flow was stopped to allow 10 minutes of exposure to the Ab-modified electrodes. After incubation period, unbound species are washed by flowing 0.1M KCl at 100 µL/min for 30 s, then flow was stopped and DPV was measured.

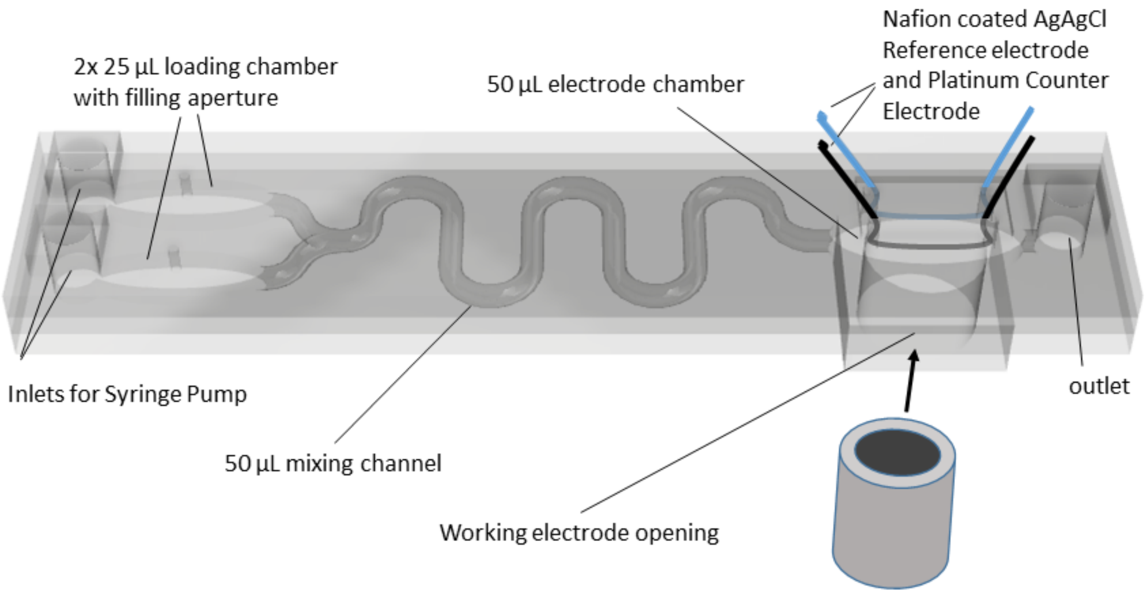

Figure S6. Design of the microfluidic chip used for sample handling and electrochemical detection. The chip houses an electrode compartment with reference Ag/AgCl and counter platinum electrodes connected to a mixing serpentine channel designed to mix sample and reagent pumped from their respective chambers. 25 µL sample and reagent are injected into their chambers through an injection hole, which is then closed using Kapton tape. Inlets are connected to a programmable syringe pump via flexible tubing

Online microfluidic CRP quantitation

Increasing concentrations of CRP spiked into 1% HS/100 mM MES, pH6.0 (as a complex protein matrix surrogate) was used to establish correlation between protein concentration and DPV signal. The microfluidic assay had an LOD of 0.1 pg/mL and a dynamic range from 0.1 pg/mL- 100 ng/mL of CRP in 1% human serum.

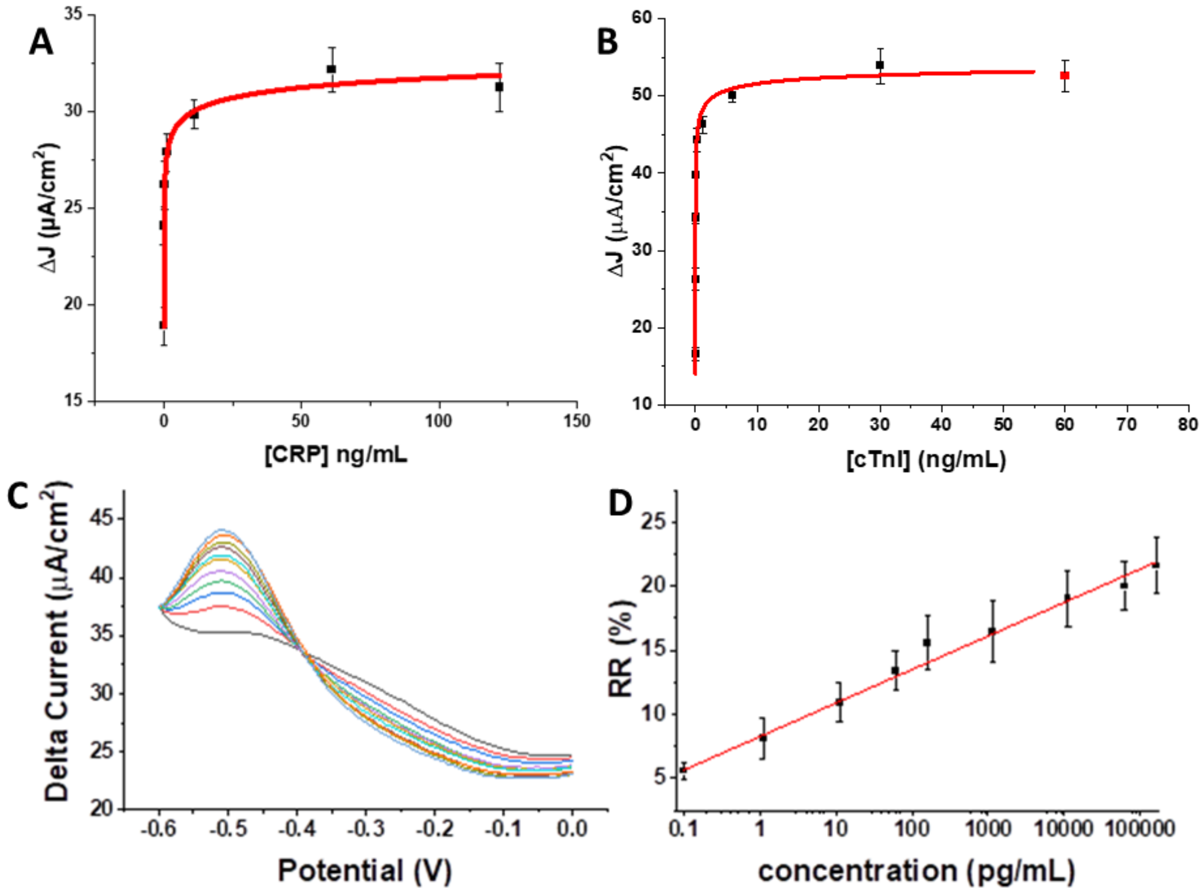

Figure S7. Representative extended calibration of singleplex assay of (A) CRP and (B) cTnI showing current saturation at concentrations beyond assay dynamic range. (C) Background corrected sample DPV curves as function of increasing concentrations of CRP spiked into 1% HS via the microfluidic cell. (D) Calibration curve plotted as relative response vs CRP concentration in 1% HS (R^2= 0.992). Data points are the average of three independent measurements on 3 different electrodes with error bars representing standard deviation.

Online assay specificity study

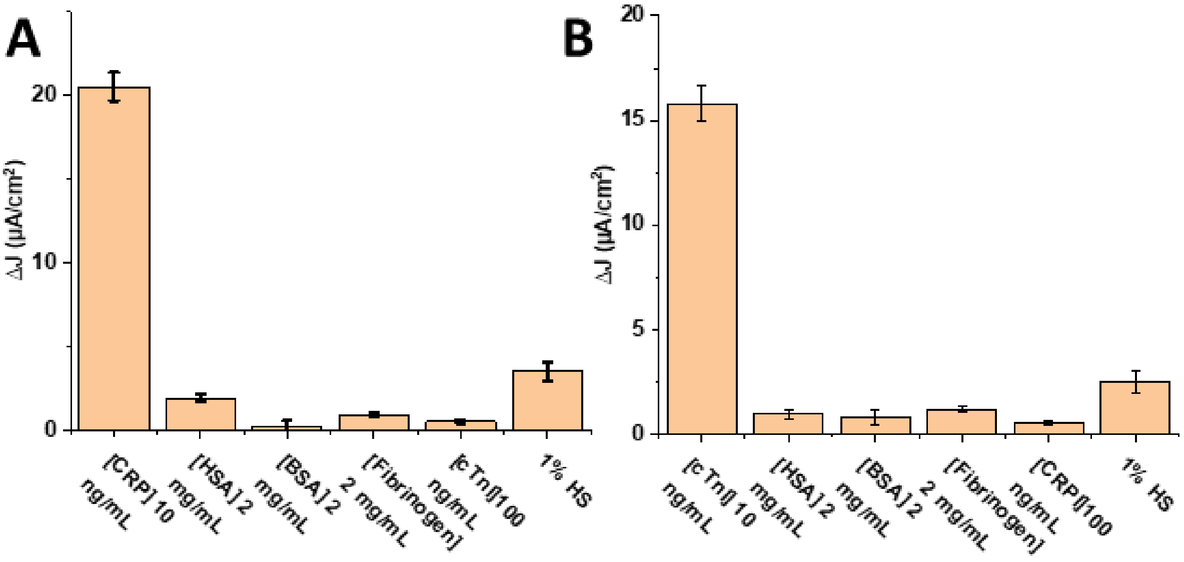

Figure S8. Electrochemical Specificity study for (A) Anti-CRP modified electrodes and (B) Anti-cTnI modified electrode in online microfluidic multiplexed protein analyses. The response to studied interfering species was between 5-15% of the target-specific signal indicating a good specificity and low cross-reactivity towards other proteins. Error bars represent standard deviation from two independent measurements.

Representative DPV peaks from on-line multiplexed protein analysis

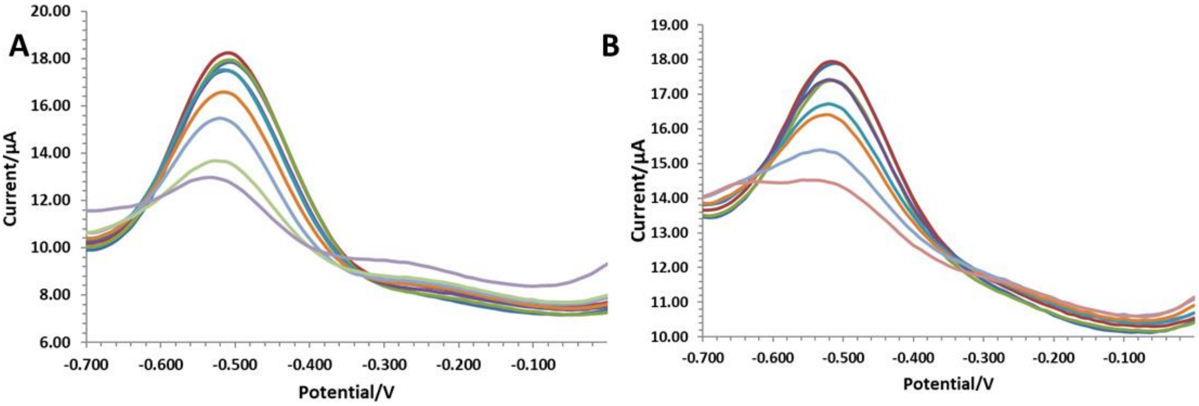

Figure S9: Representative DPV peaks for multiplexed on-line assay for CRP and cTnI as function of (A) increasing CRP connection on Anti-CRP decorated GCE and (B) cTnI increasing concentration on anti-cTnI decorated GCE.

Assay validation

The assay was validated by calculating the percent recovery of CRP spiked in 100% human serum. The electrochemical signal from un-spiked 100% human serum was measured and its CRP contents were estimated. Tested human serum was estimated to have 2.35 ng/mL of CRP; well within its normal expression range. Subsequently, two different concentrations of CRP were spiked and the recovered concentrations were calculated using the calibration data obtained in 1 % human serum (Figure 1). Spiked human serum samples showed percent recoveries between 80-120% of the spiked concentrations (Table 1) well within the acceptable range set by FDA guidelines for bioanalytical methods.[1]

Table S1: Percent recoveries from 100% human serum spiked with 0.25 and 1.0 ng CRP.

Sample	Recovered Conc.	Percent Recovery
**100% Human serum**	2.35	---------
**250 pg in 100% Human serum**	294 pg	117 %
**1.0 ng in 100% Human serum**	0.92 ng	92 %

Patient sample analyses

12 patient samples provided by the Oxford University hospital were analyzed using the proposed shotgun protein labelling assay and estimated concentrations of CRP and cTnI were compared to results reported estimated using commercial immunoassay platform (Abbott Architect). Both CRP and cTnI analyses showed excellent agreement across the two techniques.

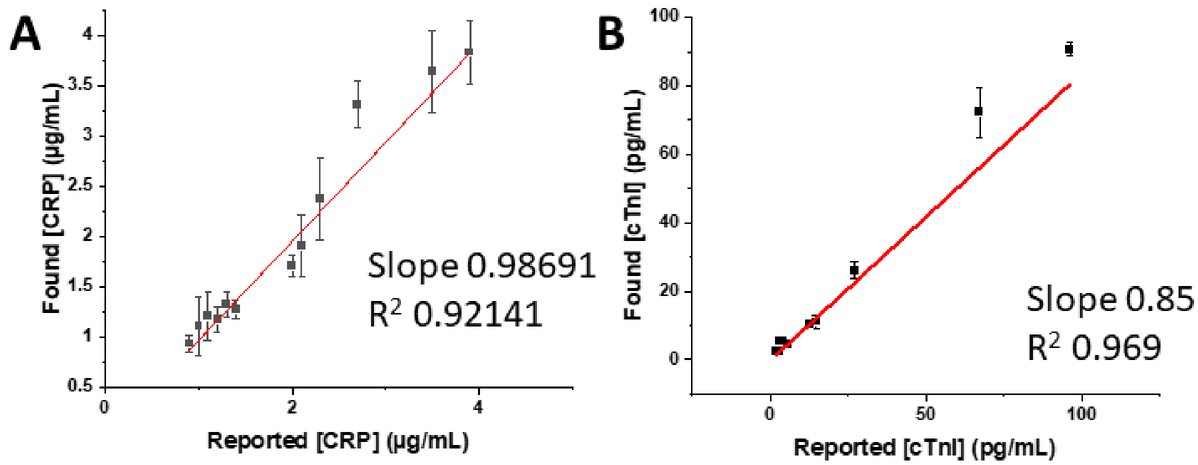

Figure S10: Correlation plots between found (A) [CRP] and (B) [cTnI] from 12 patient samples against standard immunoassay.

Electrochemical study of antibody immobilization

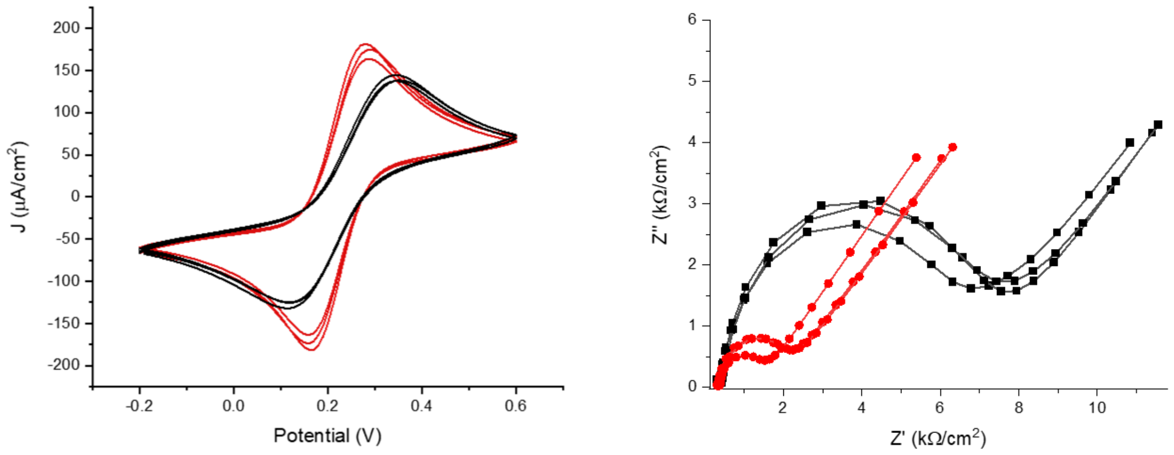

Figure S11: Electrochemical interrogation of glassy carbon electrodes before (red circles) and after (black squares) physisorption of CRP antibodies in 5mM $[Fe(CN)_6]^{4-/3-}$. Left: cyclic voltammograms recorded with scan rate of 100mV/s. Right: EIS measurements recorded at potential of 0.215V within a frequency range of 0.1 to 100kHz.

Electrochemical study of free and MB-Tagged proteins adsorbed on bare GCE

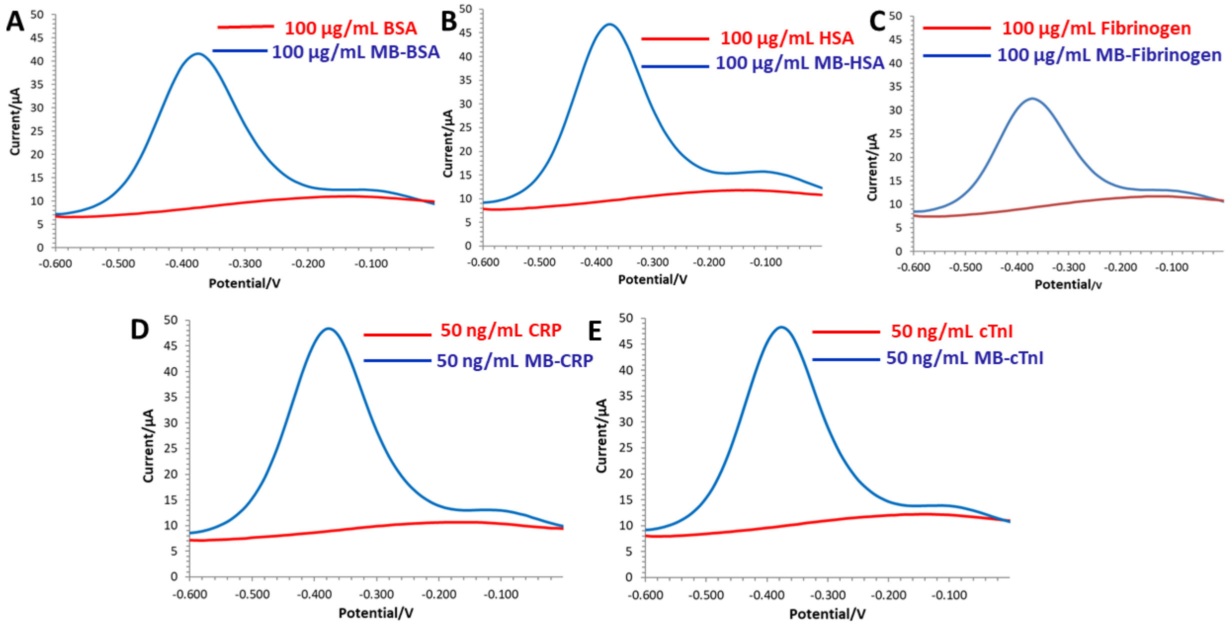

Figure S12: Electrochemical behavior of adsorbed proteins on bare unmodified GCE electrodes. Electrodes were exposed to the free protein or MB-Tagged proteins for 15 min, washed and DPV signals for each electrode were recorded. DPVs for (A) BSA and MB-BSA, (B) HSA and MB-HAS, (C) Fibrinogen and MB-Fibrinogen, (D) CRP and MB-CRP, and (E) cTnI and MB-cTnI showed that none of these proteins exhibited any inherent electrochemical activity and all are successively labelled with MB after incubation with MB-NHS.

References

(1) Viswanathan, C. T.; Bansal, S.; Booth, B.; DeStefano, A. J.; Rose, M. J.; Sailstad, J.; Shah, V. P.; Skelly, J. P.; Swann, P. G.; Weiner, R. Quantitative Bioanalytical Methods Validation and Implementation: Best Practices for Chromatographic and Ligand Binding Assays. *Pharm Res* **2007**, *24* (10), 1962–1973. https://doi.org/10.1007/s11095-007-9291-7.
